# An Introduction to the Objective Psychophysics Toolbox

**DOI:** 10.3389/fpsyg.2020.585437

**Published:** 2020-11-02

**Authors:** Thomas Hartmann, Nathan Weisz

**Affiliations:** Centre for Cognitive Neuroscience and Department of Psychology, Paris-Lodron Universität Salzburg, Salzburg, Austria

**Keywords:** open source software, MATLAB, psychophysics, EEG, MEG, fMRI, reaction time, stimulus presentation

## Abstract

The Psychophysics Toolbox (PTB) is one of the most popular toolboxes for the development of experimental paradigms. It is a very powerful library, providing low-level, platform independent access to the devices used in an experiment such as the graphics and the sound card. While this low-level design results in a high degree of flexibility and power, writing paradigms that interface the PTB directly might lead to code that is hard to read, maintain, reuse, and debug. Running an experiment in different facilities or organizations further requires it to work with various setups that differ in the availability of specialized hardware for response collection, triggering, and presentation of auditory stimuli. The Objective Psychophysics Toolbox (o_ptb) provides an intuitive, unified, and clear interface, built on top of the PTB that enables researchers to write readable, clean, and concise code. In addition to presenting the architecture of the o_ptb, the results of a timing accuracy test are presented. Exactly the same MATLAB code was run on two different systems, one of those using the VPixx system. Both systems showed sub-millisecond accuracy.

## Introduction

Writing the source code for an experimental paradigm is one of the most crucial and critical steps of a study. Open-Source software applications and libraries like PsychoPy (Peirce, 2007, 2009; Peirce et al., 2019) and the Psychophysics Toolbox (PTB; Brainard, 1997; Pelli, 1997; Kleiner et al., 2007) have enabled researchers to develop experiments using Python or MATLAB (The MathWorks Inc.), both popular programming languages within the scientific community. Both PsychoPy and the Psychophysics Toolbox essentially provide an interface to the underlying hardware, like the graphics card, the sound card, input devices (keyboards, mice, and button boxes), and devices capable of sending triggers. The libraries are also designed to ensure minimal latencies and – even more important – minimal jitter, which is a crucial requirement for recording high-quality data.

The Psychophysics Toolbox in particular has been used by generations of researchers, acting as a thin interface between MATLAB and various low-level libraries. Visual stimuli are drawn via OpenGL (Woo et al., 1999), which is also used to control and return the time the stimuli appear on screen. Auditory stimuli are controlled via PortAudio (Bencina and Burk, 2001) by directly interacting with audio-buffers. Responses are acquired via low-level access through Java. Besides standard PC hardware, the Psychophysics Toolbox supports a wide variety of specialized hardware like various eye-trackers as well as response and triggering devices – each via a unique interface. This low-level approach makes the Psychophysics Toolbox extremely versatile and flexible.

However, this low-level approach makes it harder to create source code that is easy to write, maintain, and understand. The high level of knowledge required when interacting with the hardware on this level increases the likelihood of errors in the code, while at the same time decreasing the likelihood of errors being identified. It also increases the amount of code that is necessary to accomplish a task. These aspects also make it quite difficult to learn how to use the software efficiently and correctly.

The “Objective Psychophysics Toolbox” (o_ptb, available at https://gitlab.com/thht/o_ptb) is an open-source library for MATLAB that attempts to overcome the aforementioned issues by providing an abstraction layer on top of the Psychophysics Toolbox as well as other libraries for specialized hardware. It was designed to provide a generic, enhanced, and intuitive interface to the underlying hardware while maintaining the versatility of the Psychophysics Toolbox. In this article, we will discuss common challenges and best practices for the development of an experiment and how these influenced the design of the o_ptb. In order to illustrate the benefits of the o_ptb, we wrote source-code for a simple task, including visual and auditory stimulus presentation, triggering and response collection that runs with and without the VPixx system. The first version uses the o_ptb while the second version does not (see Supplementary Materials 1, 2). Throughout this article, sections of these two files will be used to illustrate individual aspects of the design and implementation of the o_ptb. To complement the description of the toolbox, the timing properties (accuracy and jitter) will be presented for two common hardware setups.

## Software Design Goals of the o_ptb

During the planning stage of the o_ptb, we committed to the following software design choices:

### Write Code for Humans, not Computers

The major design goal of the o_ptb is that its “target audience” is human beings, irrespective of previous software development training or experience, and not computers. This is a fundamental and important distinction. Code that is optimized for the interpretation and execution by a computer describes *how* something is done (e.g., load two values into CPU registers, call multiplication method, transfer result back to RAM). Code that is optimized for human brains, on the other hand, describes *what* is done (multiply two numbers). In other words, the human perspective is a high-level one, while the computer perspective is a low-level one. The PTB, as already discussed, uses a low-level approach, making it very powerful and versatile but at the same time requiring the user to take on the “*how*” rather than the “*what*” perspective.

**Figure 1:**
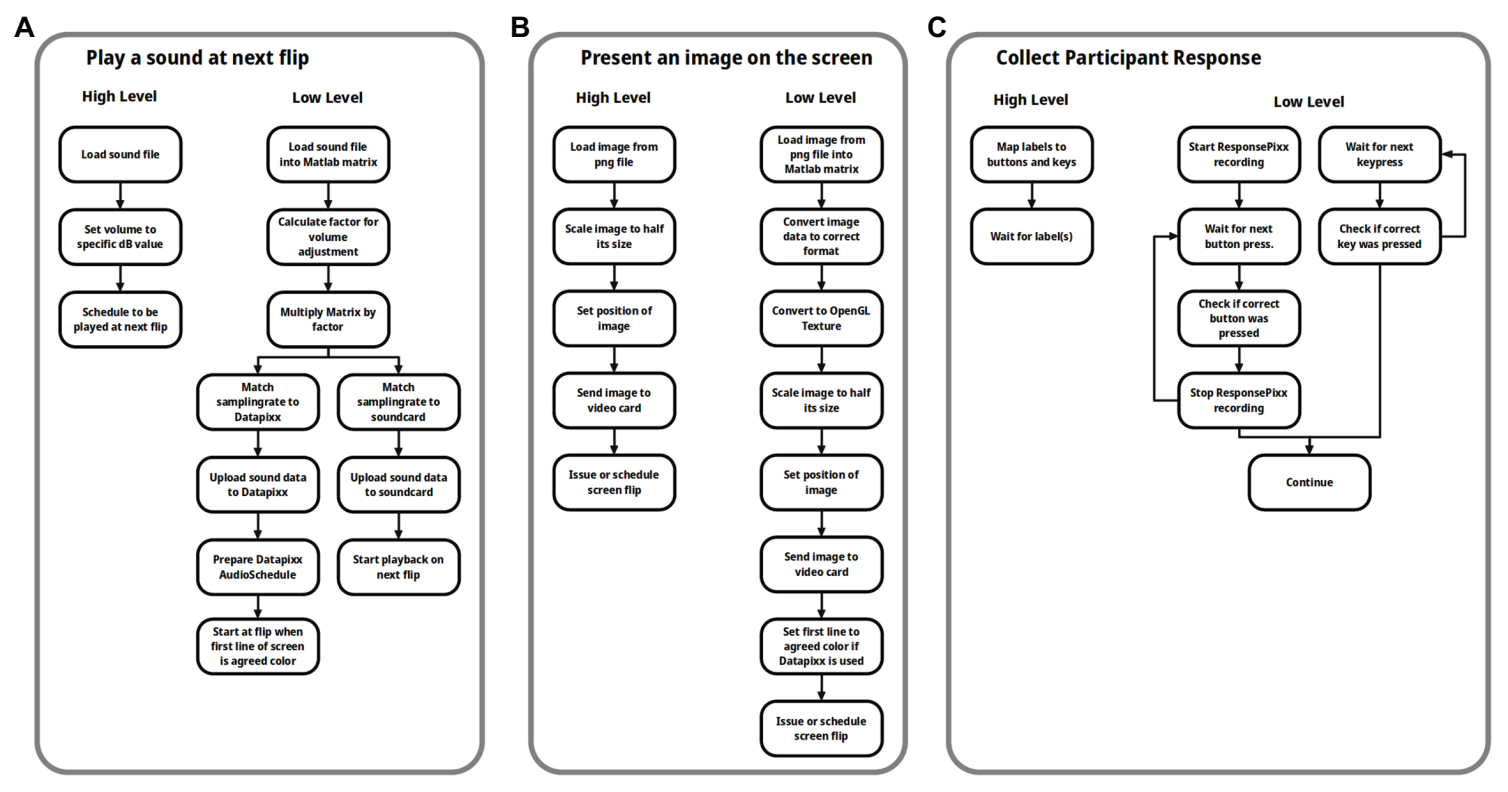
Flowchart of three common tasks from an experiment, employing a high level and a low level perspective. In all three cases: **(A)** play a sound at the next screen flip initiated by the user; **(B)** load and present an image on the screen; and **(C)** collect responses via the keyboard or dedicated device. Using the high level approach requires fewer steps which are also more intuitive.

The o_ptb, on the other hand, was designed as a high-level toolbox, allowing the user to specify *what needs to be done* and leaving the *how it is done* to the toolbox. Figure 1 illustrates this difference using common tasks from an experimental paradigm: the right hand side of each panel lists the necessary low level steps to accomplish the respective task. The purpose of the o_ptb is to enable the user to specify only the more descriptive and in total fewer steps on the left hand side of each panel. The “job” of the o_ptb is to translate these high level commands to the low level commands of the PTB.

A toolbox designed for humans must also take into account that errors in source code are common (Boehm and Basili, 2005). Many of those do not show up as an error message and stop the execution of the script. In the context of a neuroscientific/psychological experiment, examples include mistakes in formulas processing a stimulus, errors in response collection/processing or problems converting data coming from or going to the underlying hardware. These errors are dangerous as they might lead to severe problems, for example, concerning the temporal accuracy, the randomization of stimuli and trials or properties of stimuli, like their size, position, color, or volume. A well designed high level interface reduces complexity of the source code and provides well-tested code that can be reused. These factors also increase the readability of the source code. All three of these factors have been shown to reduce the number of coding errors (Lim, 1994; Aggarwal et al., 2002; Boehm and Basili, 2005; Buse and Weimer, 2010; Börstler et al., 2016). Additionally, a high level interface, like the o_ptb, can set sensible defaults that can be optimized to the current hardware configuration and run automatic consistency checks on parameters provided by the user.

### Write Once, Run on Every Hardware

It is a common scenario that computers used for stimulation are equipped with specialized hardware. This includes eye-trackers, triggering devices, response boxes, sound equipment, tactile stimulators, etc. These devices are not available on the computer that is commonly used to develop the experiment. Another scenario is that the same experiment should be conducted in different laboratories with different equipment. As stated before, the Psychophysics Toolbox supports a wide variety of general purpose as well as specialized devices. But the commands to control these different devices are specific to the hardware attached. If, for instance, one lab is equipped with the VPixx system (VPixx Technologies Inc., Saint-Bruno, Canada), sound and triggers must be controlled via the “Datapixx” library. It is also necessary to perform additional initialization steps at the beginning of the experiment. Another lab might be equipped with a high-end soundcard for auditory stimulation and a Labjack device (Labjack Corporation, Lakewood, United States) for triggering. In this case, sound needs to be controlled via PortAudio (Bencina and Burk, 2001) while triggers are controlled via the library specific to the device. The experiment is most probably developed and tested on an ordinary desktop computer or laptop without any specialized equipment.

If only the PTB is used, the user would face the following two challenges: (1) the experiment would need to determine what devices are connected to the computer and execute the code specific to the hardware found and (2) in-depth knowledge about how the devices work and differ, sometimes on a low level, is needed to ensure a reliable and correct experiment.

A high level toolbox should enable the user to write source code that is as agnostic as possible to the underlying hardware. This not only increases the flexibility of the source code, but also reduces the amount of code and its complexity, which leads to higher quality, less error prone code (Lim, 1994; Aggarwal et al., 2002; Boehm and Basili, 2005; Buse and Weimer, 2010; Börstler et al., 2016).

### Maintain the Flexibility of the Psychophysics Toolbox Whenever Possible

One of the many strengths of the Psychophysics Toolbox is its high flexibility. The user can freely interact with the underlying system without any constraints imposed by the toolbox. A high level toolbox like the o_ptb naturally trades some of this flexibility in order to decrease complexity and increase readability. However, the tradeoff between flexibility and ease-of-use must be balanced carefully. If too many constraints are imposed by the toolbox, it might be unusable for certain tasks. If the complexity remains too high, the toolbox would be of very limited benefit.

We thus chose to maintain the flexibility of the Psychophysics Toolbox whenever possible by imposing only constraints that are necessary in order to reach the other two main design goals.

## Implementation of the Design Goals in the o_ptb

The following implementation principles are direct consequences of the design goals discussed in the previous section.

### Organize Hardware and Devices Into Logical Subsystems

As already discussed, the design and workflow of the o_ptb is optimized for the way humans think and specifically how they conceptualize the organization and workflow of an experimental paradigm. Usually, an experimental paradigm can be broken down to concrete tasks like: display an image at a certain size at a certain location, play a certain sound 200 ms later and then wait for the participant to respond with option A or B. It is important to note that “low-level” aspects like the specific video or sound device and how stimuli are transmitted to these is irrelevant for the human perspective. In this “human way of thinking,” it is only relevant that visual stimuli get displayed, auditory stimuli are played and responses are collected. It is not important how the individual devices achieve the tasks.

**Figure 2:**
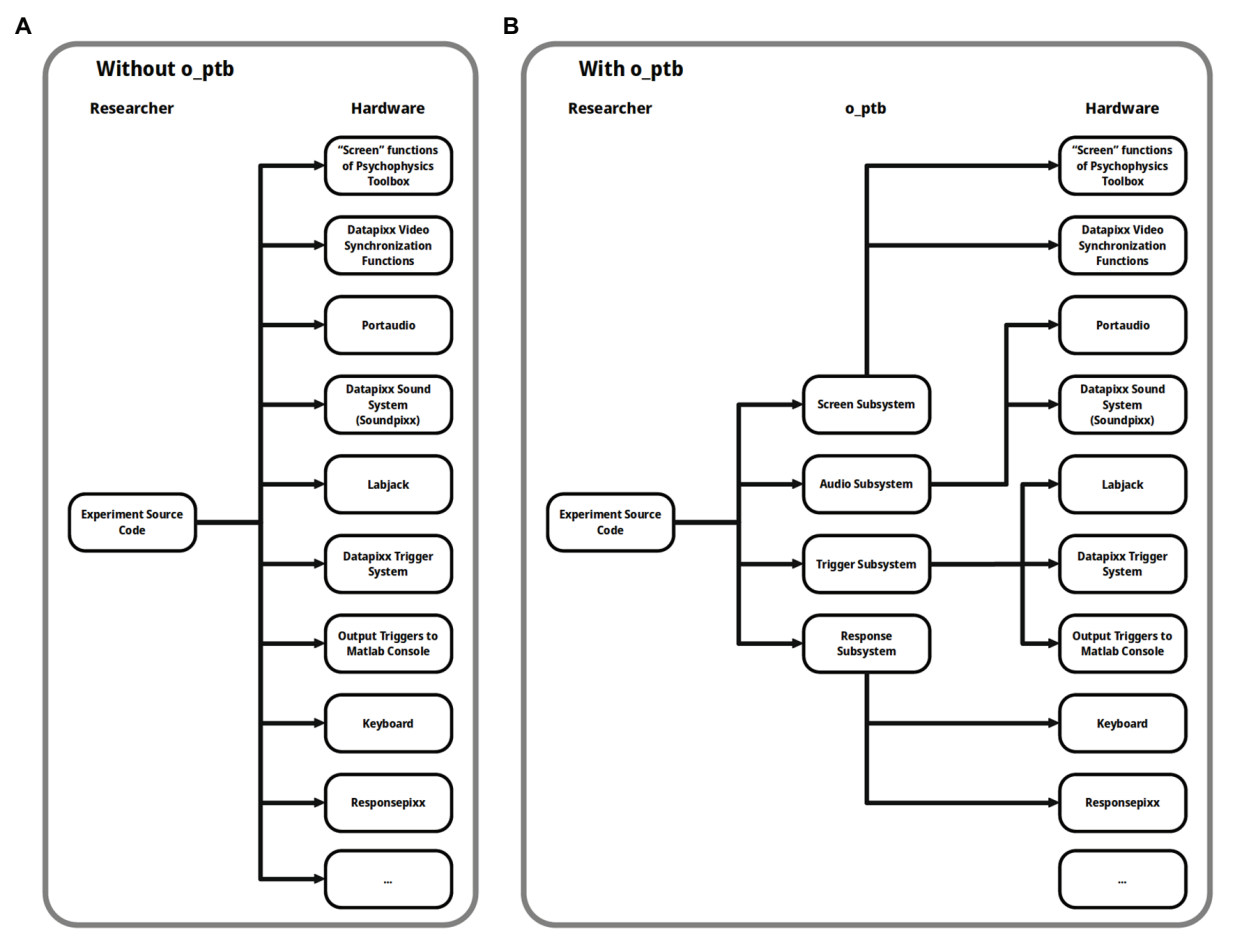
**(A)** Without the Objective Psychophysics Toolbox (o_ptb), the source code needs to access the underlying hardware directly and also needs to use different code depending on the hardware that is available. **(B)** The o_ptb provides a layer between the source code from the experiment and the underlying hardware so the source code can focus on what should be done and not how it is done for a specific hardware configuration.

The o_ptb thus provides access to so-called subsystems (see Figure 2). Each subsystem represents a modality (visual, auditory) or high level task category (triggers, responses) as described above. The respective subsystem automatically forwards the commands to the underlying device, taking care of any necessary conversions.

Currently, the o_ptb includes the following subsystems:

#### The Visual Subsystem

**Figure 3:**
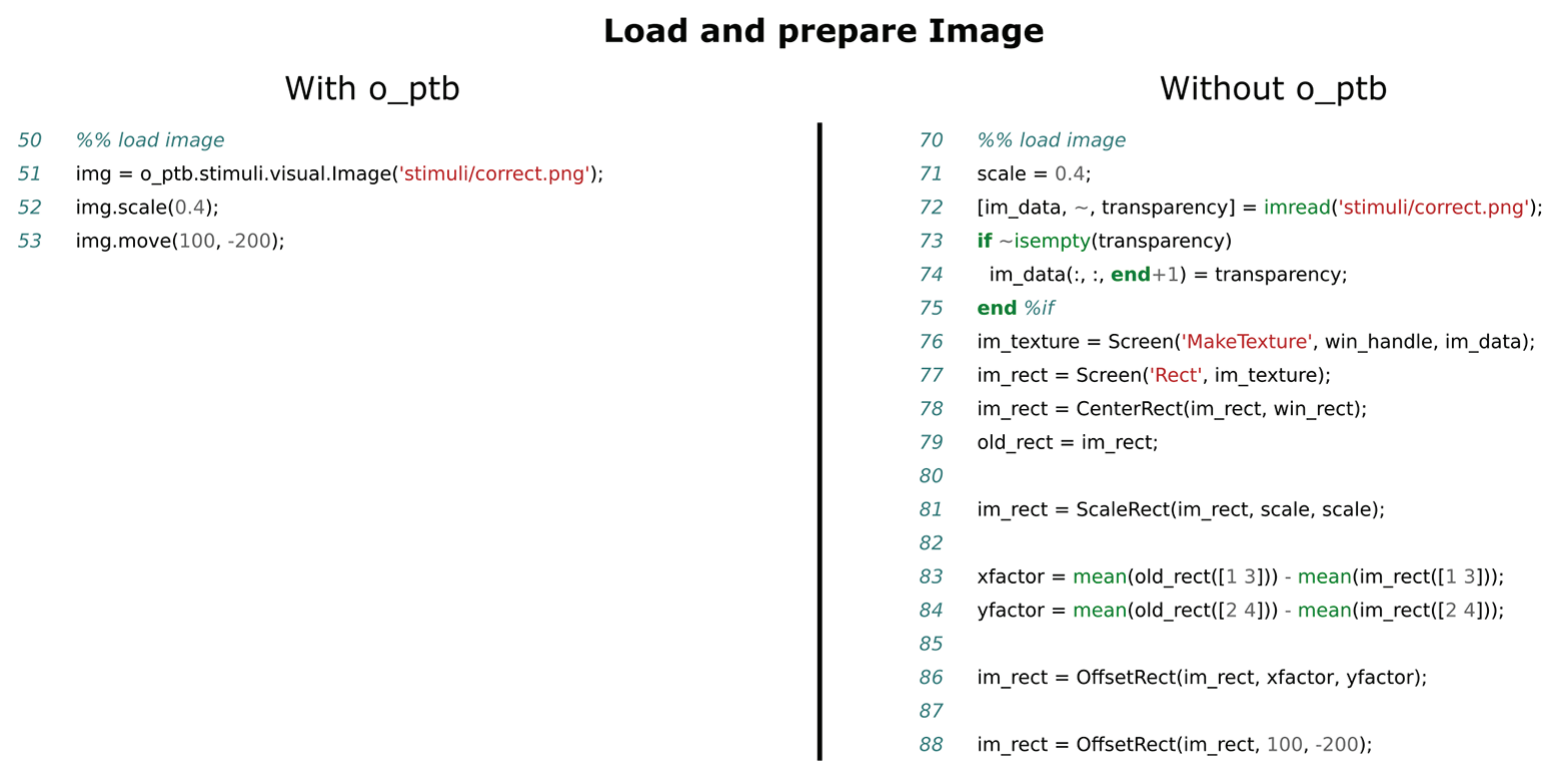
Comparison of source code needed to load an image file from disk, scale and move it. The left panel shows the source code using the o_ptb, the source code in the right panel has the same result but does not use the o_ptb.

The visual subsystem provides only a thin layer on top of the PTB. The user can choose to continue using the familiar “Screen” command, which facilitates porting of pure PTB code. Additionally, the o_ptb offers carefully designed classes for common visual stimuli like images, movies, and geometric forms. These classes provide a consistent interface to the user to create, position, scale and otherwise manipulate the stimuli (see Figure 3 for an example how to load and process a visual stimulus and Figure 5 for an example how to present the stimulus synchronously with an auditory stimulus and a trigger).

**Figure 4:**
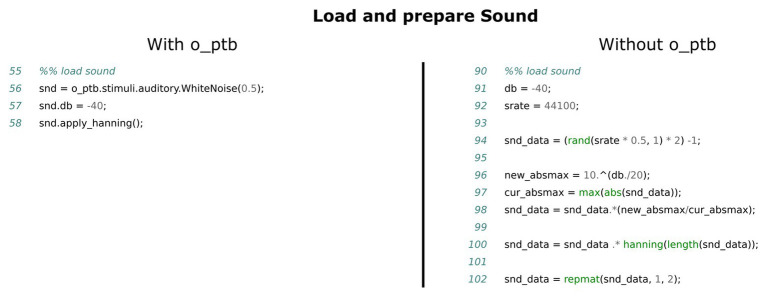
Comparison of the source code needed to create a white noise stimulus, attenuate it to −40 dB and apply a hanning window to it. The left panel shows the source code using the o_ptb, the source code in the right panel has the same result but does not use the o_ptb.

**Figure 5:**
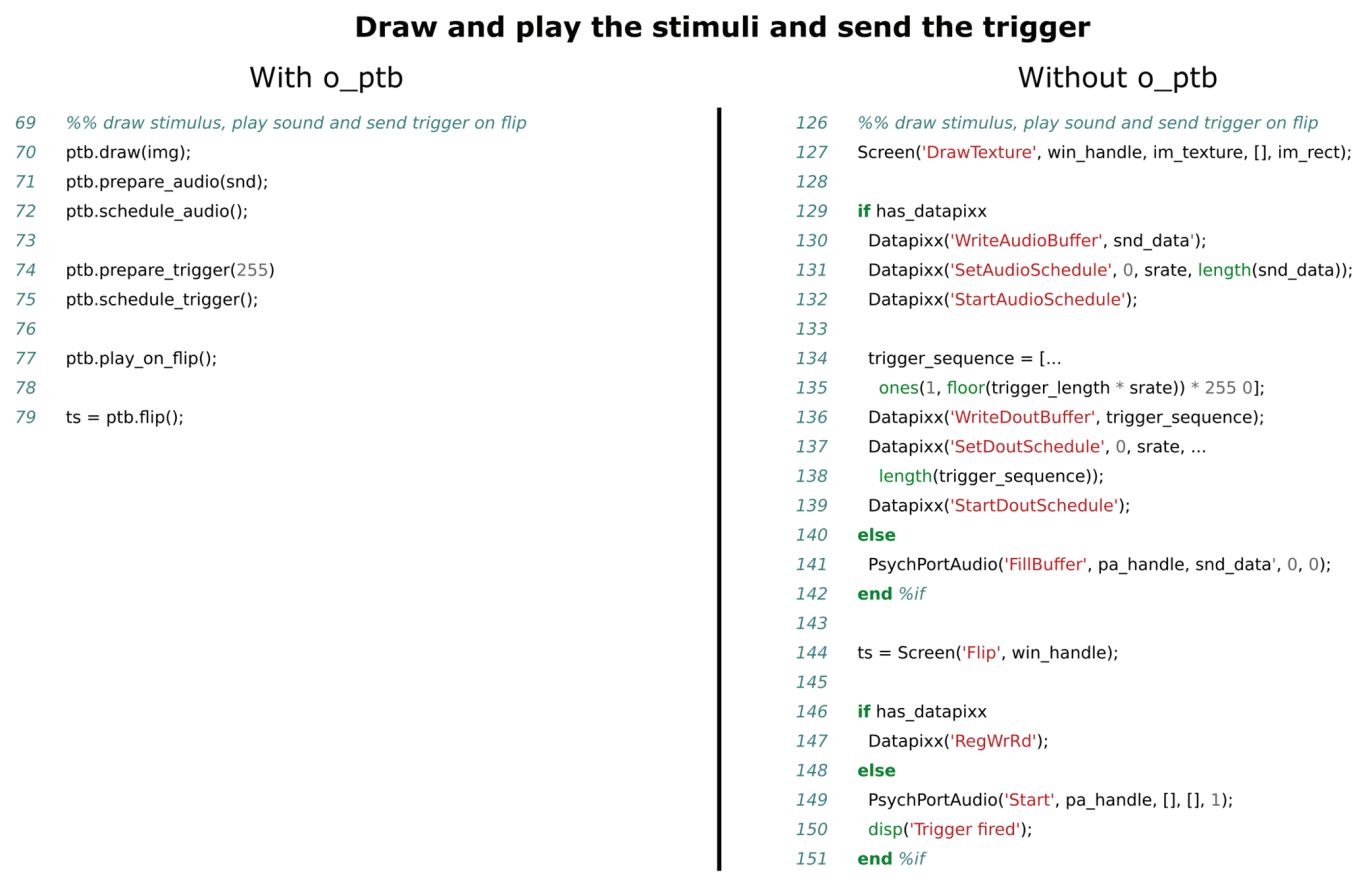
Comparison of source code needed to present the visual and auditory stimuli and send a trigger. The left panel shows the source code using the o_ptb, the source code in the right panel has the same result but does not use the o_ptb.

Behind the scenes, o_ptb takes care of using the best available method of synchronizing the visual subsystem with the other ones, taking into account the hardware configuration used (see Figure 2 for details).

#### The Audio Subsystem

The low-level commands to control sound hardware as well as the format of the data that it can process vary a lot between devices. It is thus not possible to design a common, transparent, hardware-agnostic interface, while still allowing users to use the low-level commands provided by the Psychophysics Toolbox. Instead, the o_ptb provides a set of audio stimulus classes providing the capability to load a stimulus from a wav or mp3 file, use the content of a MATLAB matrix or generate white noise or a sine wave. These sounds can be processed in numerous ways, including frequency-filtering, windowing, and vocoding (see Figure 4). The sound objects can then be scheduled to play at a certain point in time. This reference time can either be the next explicit screen flip to ensure maximum audio-visual synchronization (see Figure 5). Alternatively, the user can start the sounds at any suitable time.

#### The Trigger Subsystem

The trigger subsystem faces similar challenges like the audio subsystem. The variety of hardware devices is quite high and the low-level interface to those is highly diverse. From a high-level perspective, on the other hand, the requirements to the interface to the trigger subsystem are quite similar to those of the audio subsystem. Both basically consist of emitting an event at a specified point in time. This is reflected in the design of the trigger subsystem of the o_ptb. The user specifies what trigger value should be emitted at what delay referenced to either the next explicit screen flip or the execution of the same method used to start playing the sound objects (see Figure 5).

#### The Response Subsystem

**Figure 6:**
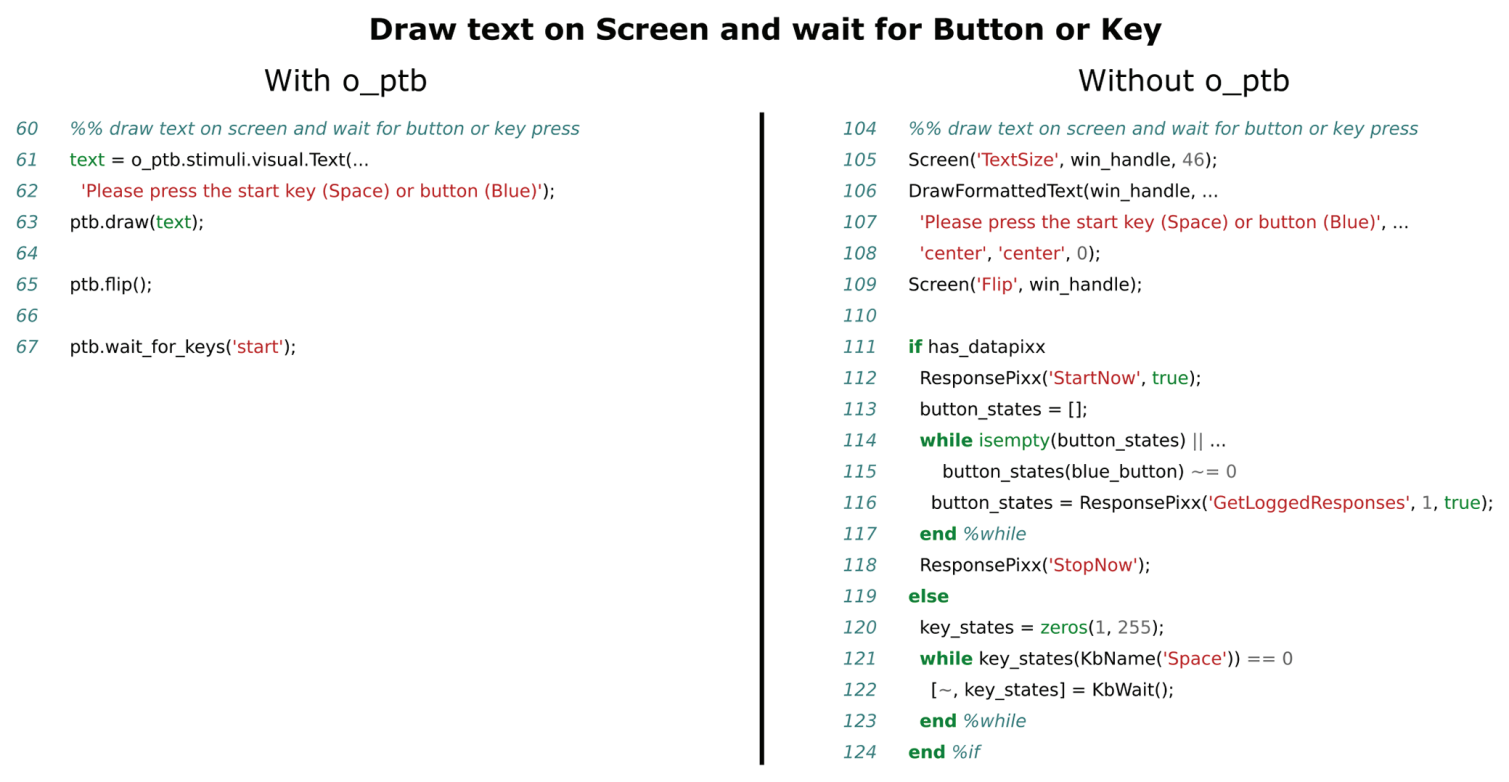
Comparison of source code needed to display a prompt and wait for the participant to press a button or key, depending on whether a VPixx/ResponsePixx system is available or not. The left panel shows the source code using the o_ptb, the source code in the right panel has the same result but does not use the o_ptb.

**Figure 7:**
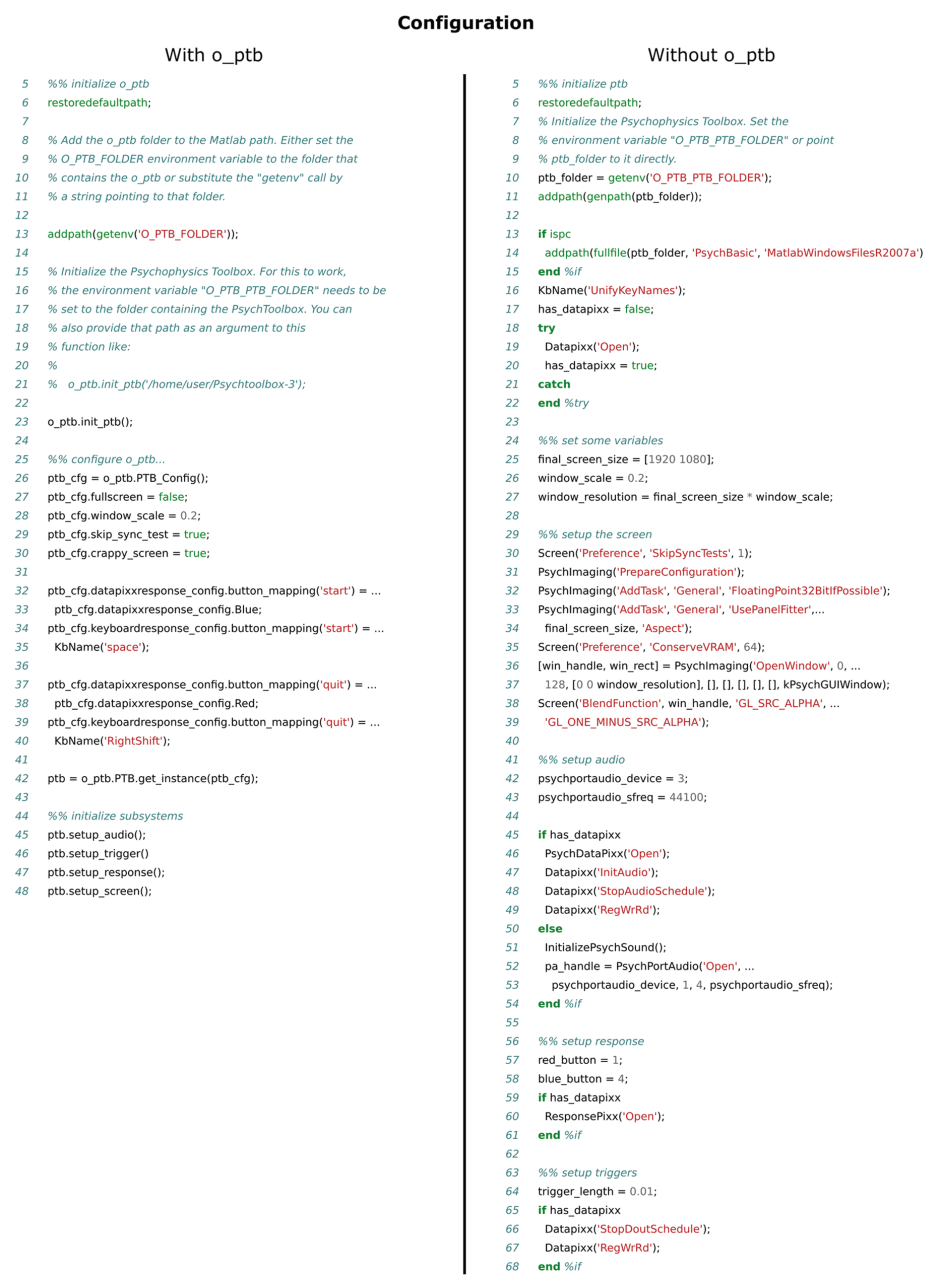
Comparison of the source code needed to configure the Psychophysics Toolbox (PTB) as well as all necessary hardware. The left panel shows the source code using the o_ptb, the source code in the right panel has the same result but does not use the o_ptb.

Designing an intuitive and unified interface for response acquisition is a challenging task because the devices used differ from the perspective of the user as well as from the perspective of the participant. While a response box might have a set of colored buttons, a computer keyboard, also commonly used for this task, has keys labeled with letters, numbers, etc. In the example of a simple reaction time experiment, in which the participant would need to react to a stimulus, he/she might need to press the “Space” key if a keyboard was used or the “Red” button if a response box is used. The o_ptb solves this by mapping semantic, meaningful labels to keys or buttons. For instance, if an experiment requires the participant to answer with “Yes” or “No,” the user would map the response label “Yes” to the right arrow key on a keyboard and to the red button on the response pad. The label “No” would be mapped to the left arrow key and the green button (see code lines 32–40 in the left panel of Figure 7 for an example). It is now possible to query whether the participant has pressed “Yes” or “No” within the code without taking care if responses are physically provided via keyboard or button box (see Figure 6).

#### Additional Subsystems

Besides the aforementioned subsystems, the o_ptb includes preliminary support for eye-tracking hardware (currently only VPixx TRACKPixx) and somatosensory stimulators (currently only CorticalMetrics).

### Stimuli Are Objects

One of the most common tasks of an experiment script is the presentation of stimuli. For instance, a visual stimulus might be an image or movie loaded from the hard drive, a geometric shape or a text. An auditory object might be a sine-wave, white noise, the sound-data read from a wav or mp3 file or a MATLAB matrix. After some manipulation or processing, they are either placed on the screen for a specified amount of time or played via the audio subsystem. It thus seems natural to think of stimuli as objects and an intuitive toolbox written for humans should thus match that representation. Object-oriented programming is a software development paradigm that is ideally suited for this kind of representation. Within the realm of the o_ptb, every visual and auditory stimulus is represented by an object. Visual objects can be moved across the screen and scaled (see Figure 3). Auditory objects provide methods to set the volume using various metrics, apply filters and ramps and other post-processing (see Figure 4). Finally, these objects are submitted to the o_ptb to display or play via the underlying audio device (see Figure 5). Any necessary hardware-specific processing like adjusting the sampling-rate is done internally.

One important aspect of the object-oriented approach is that a concrete stimulus class like a rectangle or a sine wave is hierarchically connected to a so-called “base” class. This base class implements all common methods. The visual base class, for instance, provides methods to scale and position the stimulus (see Figure 3). The auditory base class provides a great variety of processing methods like volume control, window functions, filters, and vocoding. These common methods are inherited by all concrete stimulus classes. This leads to two important benefits: (1) new stimulus classes only need to implement how to construct the specific stimulus. All methods of the base class are instantly available to it and (2) when a new method is implemented in the base class, all existing stimulus classes can instantly use it.

### Provide Central and Fault Tolerant Configuration With Sensible Defaults

The configuration of the devices and the environment used to run the experiment is highly crucial. Non-optimal configuration can lead to poor temporal accuracy, reduced stimulus quality and issues with connected devices and their possible interaction with each other. This is further complicated by the fact that some important optimizations rely on correct configuration, which can be different depending on the hardware used. Additionally, configuration options during development are different than when the experiment is actually run. Without the o_ptb, individual devices need to be configured using commands and configuration options specific to the respective device. The o_ptb not only consolidates the configuration of all supported devices in a single and well documented configuration structure. It also provides sensible defaults for most settings and runs plausibility checks to prevent common errors (see Figure 7).

In the remainder of this article, the o_ptb will be used to assess the temporal accuracy of two different hardware setups available at the University of Salzburg. While one of these setups provides the complete set of VPixx products for visual and auditory stimulation as well as triggering, the second system uses the internal video and soundcard of a PC running on Linux and a Labjack U3 USB device (Labjack Corporation, Lakewood, United States) for the triggers.

## General Requirements and Installation

The o_ptb is a toolbox running on MATLAB 2016b or higher (The MathWorks Inc.). It requires a current version of the Psychophysics Toolbox. It only uses functions provided by MATLAB and the PTB at its core. It thus works on Windows, Mac, and Linux. Certain devices that can be used with the o_ptb might need further software and/or drivers installed on the system. As these drivers might not be available for all platforms, some restrictions might apply.

The o_ptb is released under the General Public License 3 (GPL; Free Software Foundation, 2007). The latest version can be downloaded at https://gitlab.com/thht/o_ptb. Assuming that MATLAB and the PTB have already been installed on the computer, it is sufficient to copy the contents of the o_ptb to an arbitrary folder on the computer’s hard drive. More detailed instructions are available in the documentation at https://o-ptb.readthedocs.io/en/latest/install.html.

## Timing Accuracy Setup

In order to demonstrate the capabilities and limitations of the o_ptb and different hardware configurations, we measured latency and jitter between visual stimulation, auditory stimulation, and the trigger output. The timing was measured using the Blackbox2 Toolkit, (The Black Box ToolKit Ltd., Sheffield, UK) which is an independent device capable of measuring onset times of visual and auditory stimuli as well as triggers with sub-millisecond precision.

The paradigm was kept very simple: in each trial, a white circle would appear on the otherwise gray screen for 200 ms. At the same time, white noise was emitted for 100 ms and a trigger was sent. The inter-trial-interval was set to an average of 1 s uniformly jittered by 200 ms. Around 100 trials were recorded.

The paradigm was written using the o_ptb as introduced here running on top of the Psychophysics Toolbox (Brainard, 1997; Pelli, 1997; Kleiner et al., 2007).

The stimulation was run on MATLAB 2019b installed on Debian Buster. We tested two hardware setups:

The first was a HP 802E with a NVidia Quadro K620 graphics card running at a refresh rate of 120 Hz. This computer was attached to a VPixx System (DATAPixx2 display driver, PROPixx DLP LED Projector by VPixx Technologies, Canada). The VPixx devices were thus used for auditory stimulation and triggering as well as the synchronization of those to the visual stimulation. As the system is used to conduct experiments during which data is acquired by a magnetoencephalograph (MEG), sound was delivered via air-tubes (approximately 5.6 m length), introducing a physical delay of 16.5 ms.

The second was an HP Elitedesk 800 with a NVidia GeForce GT 730 graphics card connected to a ASUS VG258 Monitor running at 120 Hz. Sound was emitted via an Intel 8 Series C220 internal sound card. Trigger emission was done using a Labjack U3 device (Labjack Corporation, Lakewood, United States).

Despite the highly different hardware configuration of the two systems, the o_ptb allowed us to use exactly the same script on both machines.

The data were analyzed using pandas (Reback et al., 2020) running on Python 3.7. For each trial, the difference between all three onsets (visual, auditory, and trigger) was calculated. Finally, the mean and the SD for the differences of each pair were calculated.

## Timing Accuracy Results

### Setup 1 (With VPixx)

The average delay between the onset of the trigger and the onset of the sound was 16.74 ms. As mentioned above, this includes the 16.5 ms delay introduced by the sound having to travel through the air-tubes. The standard deviation was 0.065 ms.

The average delay between the onset of the trigger and the onset of the visual stimulus was 8.44 ms, i.e., the visual stimulus appeared one screen refresh later than the trigger. The standard deviation of this delay was 0.10 ms.

### Setup 2 (Without VPixx)

In this setup, the sound preceded the trigger by 0.17 ms on average with a standard deviation of 0.18 ms. The average delay between the onset of the trigger and the onset of the visual event was 1.36 ms with a standard deviation of 0.86 ms.

In comparison the accuracy of both systems is in the sub-millisecond range making them both acceptable for standard psychophysiological and neuroscientific research.

## Discussion

The development of experiments is a challenging task for a researcher. The o_ptb presented here not only simplifies this important task by providing a unified interface to hardware used in the experiment. It also adheres to proven principles like code reuse, high level of abstraction, and easy to read code that has been shown to reduce the likelihood of errors that could jeopardize the validity of the results and interpretations of a study (Lim, 1994; Boehm and Basili, 2005; Buse and Weimer, 2010; Börstler et al., 2016). This is achieved by adding an additional layer of abstraction between the source code of the experiment and the Psychophysics Toolbox (Brainard, 1997; Pelli, 1997; Kleiner et al., 2007) and further low-level libraries, like PortAudio (Bencina and Burk, 2001), the VPixx interface (VPixx Technologies, Canada), and the Labjack exodriver (Labjack Corporation, Lakewood, United States; see Figure 2). This means that the source code does not interact with the devices and their libraries directly. Instead, it interacts with the unified high-level interface provided by the o_ptb that then takes care of the low-level interaction. Additionally, the o_ptb provides stimulus classes for the auditory and visual subsystem providing common operations like frequency filters for the auditory and scaling and positioning for the visual subsystem.

The source code comparisons shown in Figures 3–7 illustrate the advantage of the high level approach of the o_ptb. The source code is shorter and less complex when the o_ptb is used because hardware differences are processed “behind the scenes.” It is also easier to read the source code and infer the intention of the user because intuitive names are used.

The MATLAB source code used to assess the accuracy of stimulus presentation (available online, see Methods) is another ideal showcase of the benefits of the o_ptb. Although both stimulation systems use different hardware – one providing a VPixx based setup, the second one running on standard PC components plus a LabJack for triggering – the exact same code was used to run the test stimulation.

The results of these tests show that both systems offer sub-millisecond accuracy. Using the VPixx system leads to a further increase of accuracy by a factor of 3–8. Yet, these results are not directly comparable because the availability of the VPixx system was not the only difference between them. Besides differences in the hardware of the computers, the most significant differences between the systems are (a) the use of a projector vs. an LCD monitor and (b) sound delivery via air-tubes. The purpose of this test was thus not to compare these two systems but to show that adequate accuracy can be achieved with and without specialized hardware as long as the configuration and the code of the experiment is of optimal quality, as ensured by the o_ptb.

After almost 4 years of active development and use in peer-reviewed studies (Hartmann and Weisz, 2019; Hauswald et al., 2020; Sanchez et al., 2020) and current pre-prints (Suess et al., 2020; Weise et al., 2020), the o_ptb has evolved into a stable, mature toolbox. It is extensively documented^1^ and includes tutorials to provide a smooth experience from the beginning.

To summarize, the o_ptb facilitates and optimizes the development of experiments, leads to better readable and more reusable source code and thus reduces the probability of errors. It enables users to create experiments that run on a variety of platforms without changes to the code and without any device specific sections in the code.

## Data Availability Statement

The datasets presented in this study can be found in online repositories. The names of the repository/repositories and accession number(s) can be found in the article/Supplementary Material.

The code of the timing experiment can be accessed at: https://gitlab.com/thht_experiments/blackbox_tester/-/tree/article and the data and the analysis code of the timing experiment can be accessed at: https://gitlab.com/thht/blackbox_tester_usbstim/-/tree/article.

## Author Contributions

TH planned, designed, and wrote the software described in the article. TH also wrote the first draft of the manuscript. NW contributed to the planning and the design of the software. NW also contributed to the final structure of the manuscript and provided extensive review and editing. All authors contributed to the article and approved the submitted version.

### Conflict of Interest

The authors declare that the research was conducted in the absence of any commercial or financial relationships that could be construed as a potential conflict of interest.

## Footnotes

^1^https://o-ptb.readthedocs.io
